# Vascularization of Patient-Derived Tumoroid from Non-Small-Cell Lung Cancer and Its Microenvironment

**DOI:** 10.3390/biomedicines10051103

**Published:** 2022-05-10

**Authors:** Joseph Seitlinger, Anasse Nounsi, Ysia Idoux-Gillet, Eloy Santos Pujol, Hélène Lê, Erwan Grandgirard, Anne Olland, Véronique Lindner, Cécile Zaupa, Jean-Marc Balloul, Eric Quemeneur, Gilbert Massard, Pierre-Emmanuel Falcoz, Guoqiang Hua, Nadia Benkirane-Jessel

**Affiliations:** 1 INSERM (French National Institute of Health and Medical Research), UMR 1260, Regenerative Nanomedicine, CRBS, 1 Rue Eugène Boeckel, 67000 Strasbourg, France; jo.seitlinger@gmail.com (J.S.); anasse.nounsi@etu.unistra.fr (A.N.); yidouxgillet@unistra.fr (Y.I.-G.); eloy.santos@etu.unistra.fr (E.S.P.); le@transgene.fr (H.L.); anne.olland@chru-strasbourg.fr (A.O.); veronique.lindner@chru-strasbourg.fr (V.L.); gilbert.massard@unistra.fr (G.M.); pefalcoz@gmail.com (P.-E.F.); g.hua@unistra.fr (G.H.); 2 1 Place de l’Hôpital, University Hospital Strasbourg (HUS), 67000 Strasbourg, France; 3 Faculty of Dental Surgery, University of Strasbourg, 67000 Strasbourg, France; 4 Faculty of medicine, University of Strasbourg, 67000 Strasbourg, France; 5 Transgene SA, 400 Boulevard Gonthier d’Andernach-Parc d’Innovation-CS80166, 67405 Illkirch Graffenstaden, France; zaupa@transgene.fr (C.Z.); balloul@transgene.fr (J.-M.B.); quemeneur@transgene.fr (E.Q.); 6 Institut de Génétique et de Biologie Moléculaire et Cellulaire (IGBMC), CNRS, UMR 7104, Inserm U 1258, 1 rue Laurent Fries, BP 10142, 67404 Illkirch Graffenstaden, France; grandgie@igbmc.fr

**Keywords:** patient-derived tumoroid, vascularization, tumor microenvironment, lung cancer

## Abstract

Patient-derived tumoroid (PDT) has been developed and used for anti-drug screening in the last decade. As compared to other existing drug screening models, a PDT-based in vitro 3D cell culture model could preserve the histological and mutational characteristics of their corresponding tumors and mimic the tumor microenvironment. However, few studies have been carried out to improve the microvascular network connecting the PDT and its surrounding microenvironment, knowing that poor tumor-selective drug transport and delivery is one of the major reasons for both the failure of anti-cancer drug screens and resistance in clinical treatment. In this study, we formed vascularized PDTs in six days using multiple cell types which maintain the histopathological features of the original cancer tissue. Furthermore, our results demonstrated a vascular network connecting PDT and its surrounding microenvironment. This fast and promising PDT model opens new perspectives for personalized medicine: this model could easily be used to test all therapeutic treatments and could be connected with a microfluidic device for more accurate drug screening.

## 1. Introduction

Cancer is one of the leading causes of death all over the world. Although almost 10 million people died from cancers in 2020, many cancers detected in their early phase could have been cured with effective treatments. With the discovery and the development of new anti-cancer drugs, the overall cancer death rate has significantly declined (32%) over the last three decades. Controversially, drug development has become more and more difficult and complex, which leads to a more than tenfold increase in the cost necessary to develop a drug to the approval stage [1]. Nevertheless, the average success rate to develop promising drugs against solid tumors remains below 10% [2].

Two-dimensional (2D) cell lines and patient-derived xenografts (PDXs) were the first models established for drug screening. Conventional in vitro drug discovery assays using 2D monolayer cancer cells are simple, fast, versatile, easily reproducible, and cost-effective as compared to animal models; however, they do not fully recapitulate the three-dimensional (3D) structure of the original tumors and do not retain the mutational profiles of their parental tumors. Due to the lack of tissue-specific architecture, these 2D cell models cannot reflect the complex microenvironment for cells encountered in tumor [3], nor the comparable sensitivity or resistance to drugs as the primary tumor. The PDX model retains the pathological and mutational spectrum and the 3D organization, but the establishment of this model is inefficient and labor intensive and usually takes several months per case, which makes it impractical to apply this model to guide precision medicine.

The in vitro three-dimensional (3D) cell culture has developed well and become promising models for drug screening in the last decade, and has demonstrated distinct characteristics in heterogeneity, plasticity and morphology (necrotic nuclei, hypoxia regions), as well as dynamic cell-cell and cell- extracellular matrix (ECM) interactions which mimic the natural tumor microenvironment (TME) [4,5,6]. The TME involved in tumorigenesis is comprised of ECM, stromal cells (such as fibroblasts, neuroendocrine cells, and immune-inflammatory cells), and lymphatic vascular networks [7,8], and is also important in directing the functional differentiation of organs and dictating the proper tissue function and structure [9]. Moreover, it is known that the TME may significantly change the susceptibility of tumor cells to drugs [10,11]. With these emerging examples of TME’s implication, TME constitutes a great therapeutic target besides tumor cells. Consequently, to reconstitute TME becomes a primordial task to understand tumor progression, metastasis and to screen anti-cancer drugs. Thus, 3D models based solely on cancer cell lines offer limited drug screening abilities and may not be fully predictive of the clinical response.

Patient-derived tumoroids (PDTs) are more advanced 3D cell culture models. They have been shown to recapitulate histological and mutational characteristics of corresponding tumors and allow for drug screening [12,13]. Poor tumor-selective drug transport and delivery is one of the major reasons for both the failure of anti-cancer drug screens and the resistance in clinical treatment [2,14]. Besides the pharmacokinetics and the pharmacodynamics of the drug, the transport of therapeutical molecules to solid tumors depends not only on the microvessel network established inside the tumor, but also on the properties of the extravascular tissue component [15]. Thus, to develop a functional microvascular network surrounding and within the tumor is fundamental for an efficient treatment.

Lung cancer is the most common cause of global cancer-related mortality; almost 25% of all cancer deaths are due to lung cancer in the US [16]. Two main forms of primary lung cancer are classified according to the cell types in which the cancer starts growing: non-small-cell lung cancer (>87% of cases, NSCLC) and small-cell lung cancer [17]. Based on the histologic characteristics, non-small-cell lung cancers can be subsequently divided into three subtypes: squamous cell carcinoma, adenocarcinoma and large cell carcinoma. Among the three subtypes of NSCLC, adenocarcinoma is the most common type detected in never-smoked patients [18].

In this study, we describe an improved model for vascularized PDTs and their microenvironment. Human lung fibroblast cells were first added to the tumor cells from NSCLC patients to form more complex PDTs to model spatial organization. The PDTs were then vascularized with primary human endothelial cells to finally be connected to a pre-vascularized matrix to mimic the real in vivo environment. Our promising results suggested that this innovative method could be further connected to the microfluidic device and be used for the screening of personalized medicine against cancer. Precision medicine in oncology will ultimately lead to improved overall survival, better quality of life, and a reduction in the medical and economic impact of these treatments.

## 2. Materials and Methods

### 2.1. Human Specimens, Tissue Preparation and PDT Formation

The study was conducted in accordance with the Declaration of Helsinki, and approved by the Ethics Committee of Grand Est, France (CNRIPH N° 20.11.12.42058). Samples were taken from patients who underwent major lung resection for localized lung cancer in the Thoracic Surgery Department of Strasbourg University Hospital. All written informed consent was obtained from patients the day before the surgery. As soon as the lung resection was performed, an adenocarcinomas sample (a 5 mm by 5 mm fragment) was freshly taken by a pathologist without interfering with the clinical pathological diagnosis. A healthy tissue sample was systematically taken from the lung parenchyma from the tumor of the same patient. These two biopsies were then immediately transported in cold culture medium to the laboratory for the formation of PDTs. After being washed with PBS three times, samples were cut into small pieces using sterile instruments, and mixed with 20 mL of digestion medium (DMEM/F12, 0.4% fungizone, 1% antibiotics, 500 µg/mL collagenase I, 25 µg/mL DNAse I, 25 µg/mL elastase, 100 µg/mL hyaluronidase) for 1 h at 37°, 5% CO2 in the incubator with agitation (300 rpm). After incubation, the suspension was passed through 70-μm cell strainers (Corning, Durham, NC, USA), and the strained cells were centrifuged at 240× *g* for 4 min, and the pellet was resuspended in 2 mL of ACK buffer (0.1 mM EDTA, 150 mM NH4Cl, 10 mM KHCO3) and incubated for 10 min at room temperature with gentle agitation. After having been centrifuged again at 240× *g* for 4 min, the pellet was resuspended in DMEM/F12 + 10% FCS. After cell counting, cells can be either frozen at −80 °C or be resuspended in DMEM/F12 supplemented with 10% FCS, 20 ng/µL bFGF, 50 ng/µL human EGF, 2% B27 and 1% N2 to directly form patient derived tumoroids.

Five thousand primary human pulmonary fibroblasts (HPF, CP3300-SC, CliniSciences, Nanterre, France) were mixed with 5000 patient derived cells from either tumor sample or healthy tissue in 150 µL of mixed medium (50% of HPF medium + 50% of DMEM/F12 supplemented with 10% FCS, 20 ng/µL bFGF, 50 ng/µL human EGF, 2% B27 and 1% N2) in each well of a 96-well round bottom Ultra-low attachment (ULA) plate (S-bio, Tokyo, Japan). PDT were photographed using EVOS TM XL Core microscope (Thermo Fisher Scientific, Bothell, WA, USA).

### 2.2. Vascularization of Fibrin Matrix and PDTs

The fibrinogen (F3879-1G, Sigma-Aldrich™, Saint-Quentin-Fallavier, France) was mixed with 2.5 × 10^5^ /mL of HPF, 1.25 × 10^5^ /mL Human umbilical vein endothelial cells (GFP-HUVECs, PB-CAP-0001GFP, PELOBiotech) and 1.25 × 10^5^ /mL human mesenchymal stem cells (hMSCs C-14092, Promocell, Heidelberg, Germany) from adipose tissue were added to fibrinogen. VEGF was added to a final concentration of 500 ng/mL (Human VEGF-165 Recombinant Protein, PHC9394, Life Technologies, Illkirch-Graffenstaden, France) and mixed gently with the fibrinogen and cells. Thrombin (T6884-1KU, Sigma-Aldrich™, Saint-Quentin-Fallavier, France) was added with a final concentration of 4IU/mL, and mixed gently with the mix of fibrinogen and cells, and quickly dropped into the culture insert (ThinCert^TM^ 12 well, pore size 1 µm, 665610, Greiner bio-one, Frickenhausen, Germany) to polymerize the fibrinogen and obtain a fibrin gel included with the cells. When the fibrin gel is polymerized, 700 µL of medium was added to the bottom of the well. Inserts were cultured in the incubator overnight and 150 µL of culture medium was added on the gel the next morning. Cells in a fibrin matrix were incubated at 37 °C in a humidified atmosphere of 5% CO2 for three days.

After three days of culturing the PDTS in the ULA plates, 4 × 10^3^ endothelial cells labeled GFP or RFP (RFP-HUVECs, PB-CAP-0001RFP, PELOBiotech) were added to each of the wells to cover the PDTs to “pre-vascularize” the PDTs and favor its vascularization. PDTs were incubated at 37 °C in a humidified atmosphere of 5% CO2 for an additional three days before the deposition on the matrix. Seven days after the deposition, the matrix with PDTs was fixed and followed as indicated in Section 2.5.

### 2.3. Combination of Vascularized Fibrin Matrix and Vascularized PDTs

After three days of separate culture of pre-vascularization for both fibrin matrix and PDTs to develop the vascularization in each compartment, the vascularized fibrin gel and PDTs were combined. Several PDTs were deposited on the fibrin matrix in the culture inserts and incubated at 37 °C in a humidified atmosphere of 5% CO2 for seven days. At day seven, culture inserts were fixed with 4% paraformaldehyde (PFA) for 20 min. Two hundred nM DAPI solution (Sigma-Aldrich, Saint-Quentin-Fallavier, France) was added for 20 min. The samples were observed under an epifluorescence microscope (Leica DM4000 B, Nanterre, France) and confocal microscope (Leica SP8x, objective HC FLUOTAR 25X/0,95, Nanterre, France).

### 2.4. Histologic Examination

Patient biopsies and PDTs were fixed in 10% neutral-buffered formalin and then processed for histologic examination including paraffin embedding, sectioning, and staining with hematoxylin and eosin. Sections from selected paraffin blocks for each specimen were used for immunohistochemical analysis. Slides of 4-um-thick tissue sections were incubated at room temperature in an antigen retrieval process (EDTA citrate buffer, pH 8.3, Cell Conditioning Solution (CC1), Ventana Medical Systems, Tucson, AZ, USA), revealed with ‘Ultra View’ Universal DAB Detection kit (Roche Diagnostics SA, Rotkreuz, Switzerland). They were treated on BenchMark ULTRA automated slide-staining instrument (Ventana Medical Systems, Tucson, AZ, USA) with the following monoclonal antibodies: pan-keratin (Clone SPT24, Novocastra/Leica, Nanterre, France), KI67 (Clone Mib-1, Dako, Santa Clara, CA, USA), CD45 (Clone 2B11+PD7/26, Dako, Santa Clara, CA, USA) and CD31 (Clone EP78, CliniSciences, Nanterre, France).

### 2.5. Immunofluorescence Staining

PDTs were fixed in Tissue-Tek^®^ OCT (Optimum Cutting Temperature, Fisher Scientific, Illkirch-Graffenstaden, France) and frozen at −20 °C. 10-μm sections made with cryostat (Leica, CM3000, Nanterre, France) were fixed in 4% paraformaldehyde for 10 min at 4 °C, washed three times with PBS and then incubated in PBS containing 1% BSA and 0,1% Triton X-100 for 30 min at room temperature (RT). After wash, sections were incubated with indicated primary antibodies: anti-CD31 (ab28364, Abcam, Paris, France), Ki67 (ab279653, Abcam, Paris, France), TTF1 (D2E8, Cell signaling) overnight at 4 °C. Primary antibodies were detected by incubating with Alexa Fluor™ 488- (A11001, Invitrogen, Illkirch-Graffenstaden, France) and Alexa Fluor™ 555-conjugated (A31572, Invitrogen, Illkirch-Graffenstaden, France) secondary antibodies for 1 h at RT. After washing, sections were incubated with Alexa Fluor™ 555-conjugated Phalloidin (A34055, Invitrogen, Illkirch-Graffenstaden, France) for 20 min at RT. Samples were washed with PBS before incubation with 200 nM DAPI (Sigma-Aldrich, Saint-Quentin-Fallavier, France) for 10 min at RT. The slides were observed under an epifluorescence microscope (Leica DM4000 B, Saint-Quentin-Fallavier, France) and confocal microscope (Leica SP8x, objective HC FLUOTAR 25X/0,95, Saint-Quentin-Fallavier, France). For the whole-mount IF analyses, except for the PBS wash, all incubations were performed overnight.

## 3. Results

### 3.1. Lung Cancer PDTs Maintain Histopathological Features of Original Cancer Tissue

**Figure 1:**
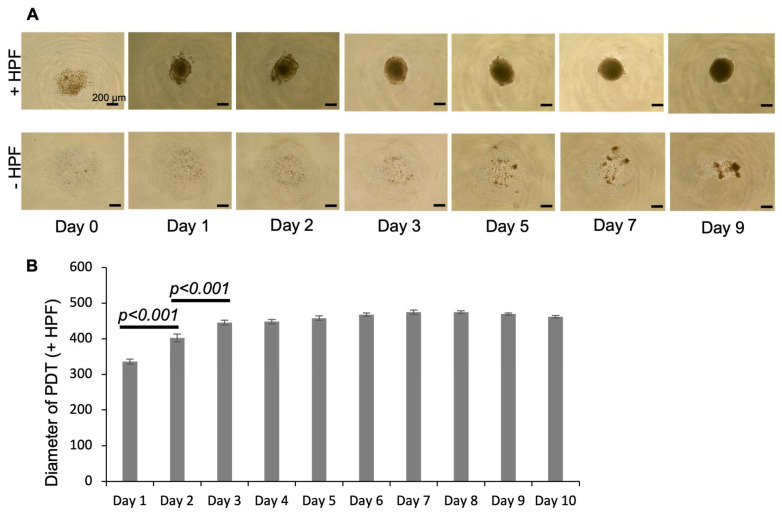
Formation of patient-derived tumoroids (PDTs). (**A**) Daily microscopic observation of the PDTs with or without HPF cells. (**B**) The diameter of 24 PDTs were measured every day using FIJI software (Version: 2.0.0-rc-69/1.52p). Measurements were repeated for PDTs formed from three biopsies.

Twenty-six lung adenocarcinoma samples were harvested for the formation of PDTs with a 100% rate of success. PDTs were formed within 24 h in a 96-well ULA (ultra-low attachment) cell plate; the size of the PDTs was significantly increased during the first three days and reached its maximum at D3 (Figure 1A,B). However, without adding human fibroblast, the PDTs could not be formed in 10 days using cells dissociated from the patient’s biopsy alone (Figure 1A).

**Figure 2:**
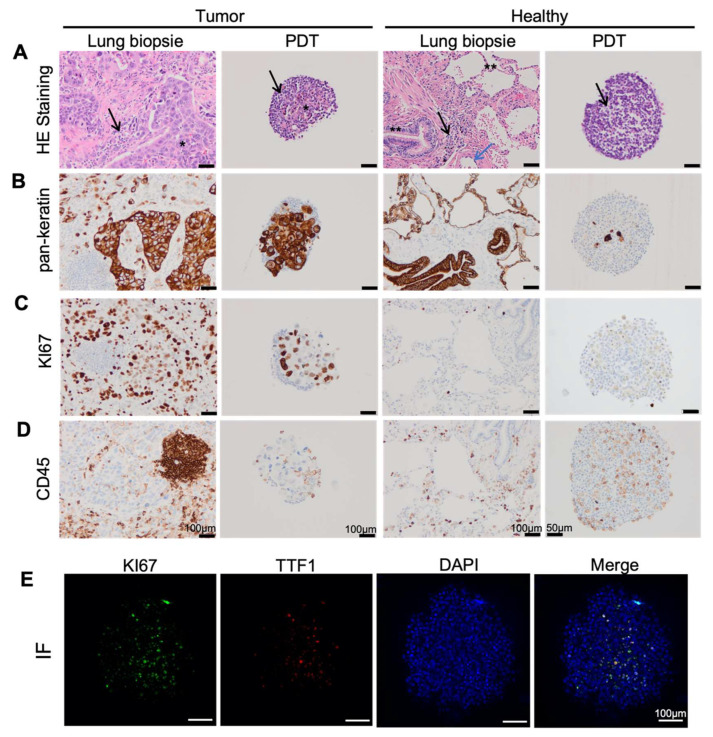
Characterization of patient-derived tumoroids (PDTs). (**A**–**D**) Immunohistochemistry analyses performed on the lung biopsy and its corresponding PDTs with indicated specific antibodies. *: epithelial tumoral cells, **: epithelial non tumoral cells (normal respiratory and alveolar epithelium), black arrow: inflammatory cells, blue arrow: endothelial contingent. (**E**) Immunofluorescence analyses performed on PDTs with indicated stainings.

In order to investigate whether the PDTs formed with cells dissociated from lung cancer biopsies maintain the histopathological features of the original cancer tissue, we compared the immunochemistry analyses performed from the frozen section with those performed directly on cancer tissue by the pathology laboratory of the hospital (histological mirror sections). Hematoxylin and eosin stains showed the invasive non-small cell carcinoma arranged in large sheets of moderate atypical cells surrounded by a fibrous stroma with inflammatory cells in tumor tissue, while bronchial and alveolar structures with conjunctive interstitial tissue punctuated by some macrophages and lymphocytes in the normal lung tissue (Figure 2A). The neoplastic cells which could cause tumor growth were found located in the center of tumor PDT with inflammatory cells in the periphery; however, this feature was not observed in PDT derived from healthy tissue (Figure 2A).

Immunohistochemistry analyses (IH) showed a stronger staining of keratins, specific markers for epithelial cells, in tumor tissue and corresponding PDTs than in healthy tissue and its corresponding PDTs (Figure 2B). Keratins were highly expressed in carcinomatous cells in lung cancer, while they mainly expressed in the respiratory and alveolar epithelium in normal lung tissue (Figure 2B). The Ki67 protein is commonly used as a proliferation marker for human tumor cells. Not surprisingly, a high expression of Ki67 was observed in lung cancer tissue and its corresponding PDTs in contrast to that observed in the normal tissue and its corresponding PDTs (Figure 2C). Tumor-infiltrating lymphocytes (TIL) have been associated with a good prognosis, especially for immunotherapy, in lung cancer [19,20]. IHC analyses using CD45 antibodies demonstrated the presence of immune cells (possibly TILs) in both cancers and healthy tissues and their corresponding PDTs (Figure 2D).

Thyroid transcription factor 1 (TTF-1) is expressed in more than 70% of adenocarcinoma, but rarely in squamous cell carcinoma. Immunofluorescence showed that PDTs formed with adenocarcinoma cells contained TTF-1 positive cells (Figure 2E). In addition, these TTF-1 positive cells expressed Ki67, indicating that these TTF-1 positive adenocarcinoma cells were proliferating (Figure 2E).

### 3.2. Endothelial Cells Infiltrate PDT from Normal Vascular Microenvironment

**Figure 3:**
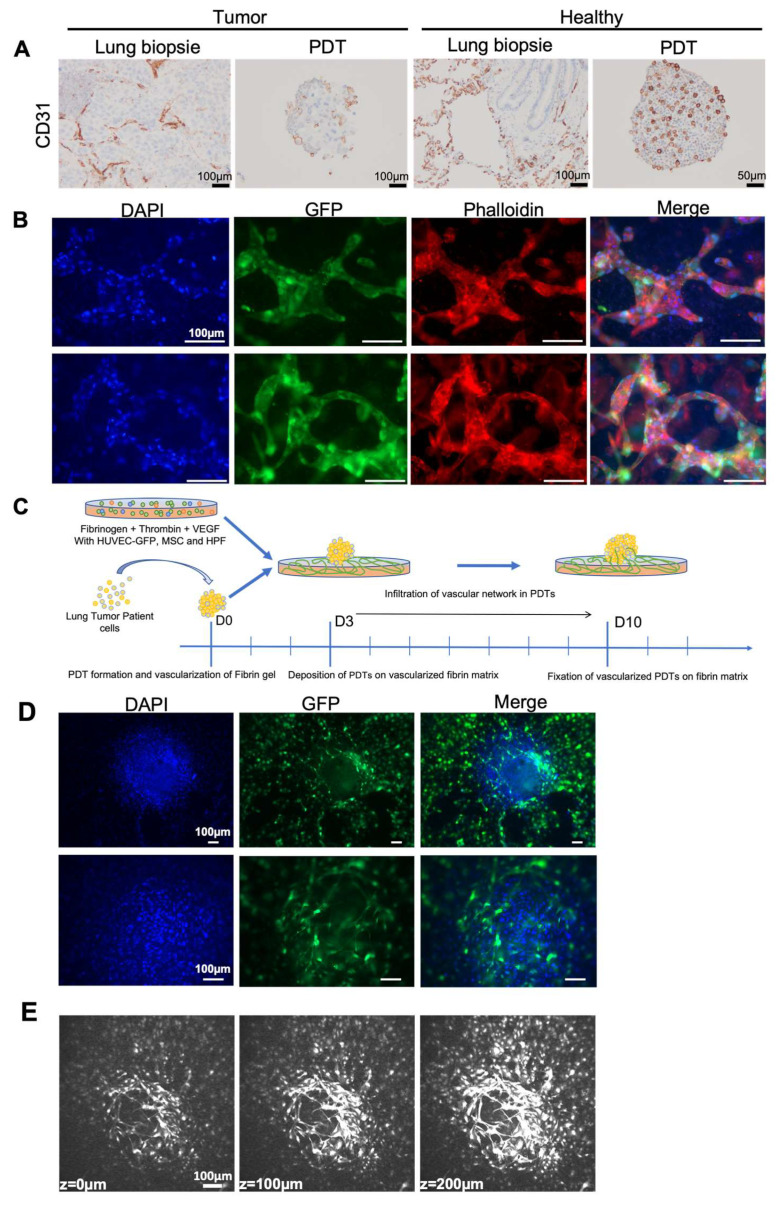
Endothelial cells infiltrate PDT from normal vascular microenvironment. (**A**) Immunohistochemistry analyses performed on the lung biopsy and its corresponding PDTs with CD31 antibody. (**B**) Immunofluorescence analyses showing the vascular network developed in fibrin gel. (**C**) Experimental scheme for the combination of vascularized fibrin gel and PDTs. (**D**,**E**) Infiltration of endothelial cells into the PDTs. (**D**) Immunofluorescence analyses; (**E**) Confocal microscopic Z-Stack analyses.

The vascular network is an important component of the tumor microenvironment. Since the tumor vasculature is significantly different from the normal vasculature which is made from well-organized and evenly distributed blood vessels, the oxygen and nutrients cannot be transported due to its immature vessel with abnormal bulges and irregular geometry, which leads to the resistance to current clinical therapies. Immunohistochemistry analyses showed the presence of CD31 positive endothelial cells in both lung adenocarcinoma samples and in healthy tissue, as well as in their corresponding PDTs (Figure 3A). Interestingly, the CD31 positive endothelial cells are found significantly more in the PDTs formed with healthy tissue than in those formed with tumor cells (Figure 3A).

We first mixed the GFP-labelled HUVEC, hMSC and HPF cells with the fibrin matrix to prepare a vascular network to mimic the normal surrounding microenvironment in vivo. As shown in Figure 3B, the vascular network was established after a three-day culture, and the diameter of vessels varied from 20 to 60 µm. We then deposited the PDT on this vascularized fibrin matrix for seven days (Figure 3C). The microvessel network developed by GFP-labeled HUVECs encompassed the PDTs (Figure 3D). Confocal microscopic analysis and 3D reconstruction confirmed that the microvessels infiltrated PDTs (Figure 3E, Supplementary Figure S1). These results clearly indicated that the surrounding vascular network could infiltrate the PDTs.

### 3.3. Endothelial Cells Infiltrate Normal Surrounding Microenvironment from Vascularized PDT

**Figure 4:**
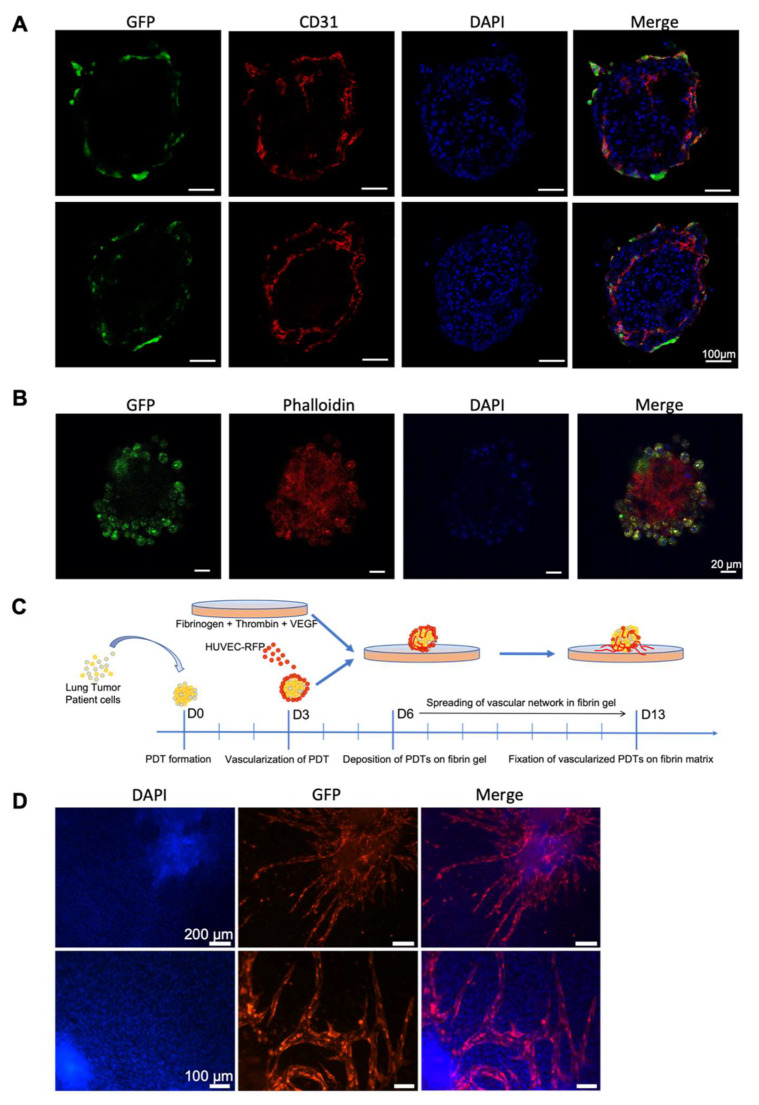
Endothelial cells infiltrate the normal surrounding microenvironment from vascularized PDT. Immunofluorescence analyses for vascularized PDTs. (**A**) cryosection staining; (**B**) whole mount staining. (**C**) Experimental scheme for the combination of vascularized PDT and fibrin gel. (**D**) infiltration of endothelial cells from PDO to fibrin gel.

In order to investigate whether endothelial cells could in reverse infiltrate the non-vascularized surrounding microenvironment from PDT, we first established pre-vascularized PDT with GFP-labeled HUVECs added to each well containing a PDT at day three. Three days after the co-incubation, PDTs were collected and fixed either in OCT for frozen section or in PFA for whole mount staining. Immunofluorescence using CD31 antibody on frozen sections revealed endothelial cells both on the peripheric and inside PDTs (Figure 4A). GFP staining confirmed that GFP-labeled HUVECs were mainly on the peripheric part of the PDTs; however, few GFP-labeled HUVECs indeed infiltrated the PDTs in three days (Figure 4B). Not all CD31 positive cells are GFP positive, thus confirming the presence of endogenous endothelial cells in the PDTs. Whole mount IF staining was further performed on the PDTs. Phalloidin staining showed the extracellular matrix of the PDT, while GFP staining confirmed the presence of infiltrated GFP-labeled HUVECs inside the PDTs. These results demonstrated that endothelial cells added to the PDT culture could infiltrate the PDTs.

Next, pre-vascularized PDTs with RFP-labeled HUVECs were placed on top of non-vascularized fibrin gel (Figure 4C and Supplementary Figure S2A). After seven days of culture, angiogenesis occurred in fibrin gel as the RFP-labeled HUVECs developed a microvascular network in the surrounding fibrin matrix (Figure 4D and Supplementary Figure S2B).

### 3.4. Vascular Network Connection between Vascularized Microenvironment and Vascularized PDT

**Figure 5:**
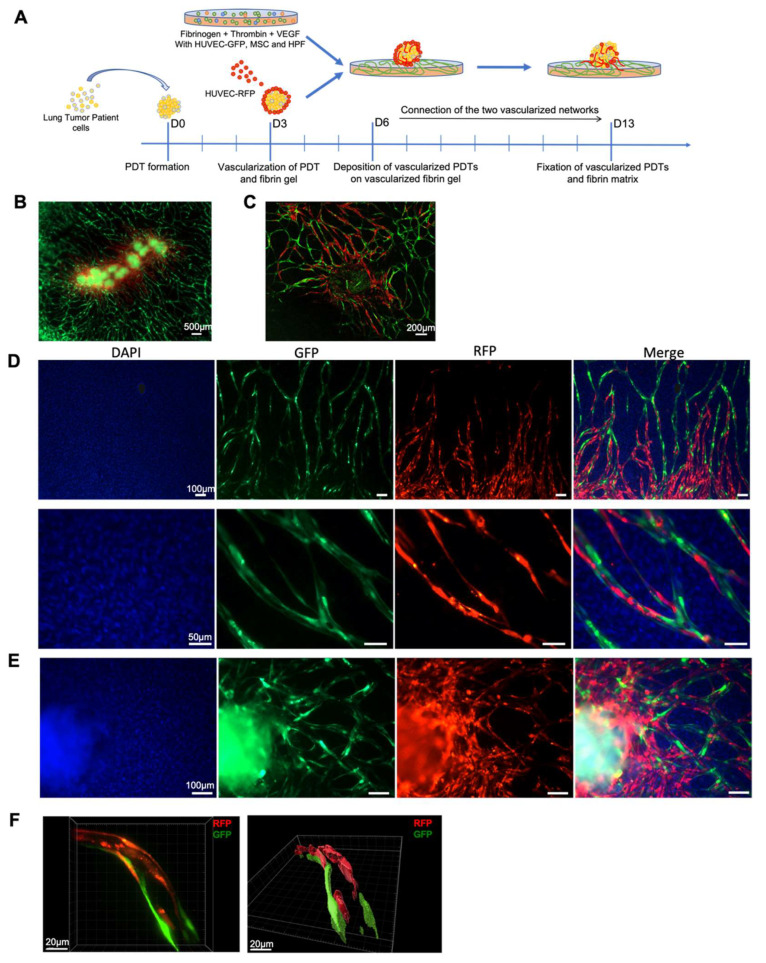
Vascular network connection between the vascularized microenvironment and vascularized PDT. Immunofluorescence analyses showing (**A**) Experimental scheme for the combination of vascularized PDTs and vascularized fibrin gel. (**B**) the global vascular network connecting PDTs and the fibrin gel; (**C**) the global vascular network surrounding one PDT; (**D**,**E**) the connection of two different original vascular networks in the fibrin gel (**D**) and inside the PDO (**E**); (**F**) 3D-reconstruction of one vessel developed by both GFP- and RFP-labeled endothelial cells.

The main purpose of this study is to connect the pre-vascularized PDT with the vascularized microenvironment. Consequently, PDTs pre-vascularized with RFP-labeled HUVECs were laid on a fibrin matrix previously vascularized with GFP-labelled HUVECs (Figure 5A). Seven days later, confocal microscopy analyses confirmed that microvessels developed by both GFP- and RFP-labeled HUVECs connected together and formed a single vascular network (Figure 5B–E), and that the red vascular network originating from PDTs extended and joined the green vascular network developed by the GFP-labeled HUVEC cells in the fibrin matrix (Figure 5B–D). Importantly, GFP-labeled HUVECs from the fibrin matrix could reach and infiltrate the PDT (Figure 5E). The 3D reconstructions obtained from the confocal microscopy analyzes confirm that these microvessels presented a lumen (Figure 5F and Supplementary Figure S3).

## 4. Discussion

Patient-derived tumoroids (PDT) or patient-derived organoids (PDO) have gained in importance over the last few years, and these models are now widely used for drug screening and anti-resistance research in various diseases, including several cancer types [12,21,22,23,24,25,26,27]. Most of the organoids used in these studies were formed in Matrigel, which supplies the extracellular matrix proteins preparation scaffold. However, this approach could not be easily standardized because the concentrations of growth factors and other biologically active components in Matrigel may vary from different references, even different batches and the overall composition remains unknown [28]. In this paper, patient-derived tumoroids were formed from NSCLC without adding an additional extracellular matrix, but only with primary human pulmonary fibroblasts which are isolated from human lung tissue. By adding the HPF cells into the culture, we increased the success rate of the PDT formation (without failure) and decreased the time needed to form PDT as compared to previously published research [29].

Personalized medicine, also called precision medicine, is not designed to create new drugs which are unique to each patient, but rather to propose the most efficient treatment according to their individual characteristics. The current 3D patient-derived organoid models have been validated for drug screens and could also be used to predict the personalized treatment [30]. These organoids are very beneficial for drug screening, gaining mechanistic insight and the creation of cryobanks for both research and clinical purposes. However, protocols using Matrigel or other matrixes usually need weeks or months to generate the patient-derived organoids, which is a drawback in giving a “back-to-patient” treatment within two to three weeks after the reception of a biopsy from the patient. We have demonstrated in this paper that the PDTs formed in this model maintain the histopathological features of the original cancer tissue (Figure 2A–D). The presence of CD 45+ cells within the PDTs indicates that tumor infiltrating lymphocytes (TILS) could be present in PDTs. Consequently, it would be interesting to test the checkpoint inhibitors (PD-1/PD-L1) with these PDTs. In addition, the preliminary results from the genomic mutation analyses demonstrated that each specific KRAS mutation from the tumor has been successfully detected in PDTs (data not shown), which suggests that our model is also suitable for screening targeted therapies. Most importantly, the vascularized PDTs could be formed in six days (Figure 1A and Figure 4A,B), allowing us to propose a “back-to-patient” personalized treatment within two to three weeks.

The most common treatments, such as chemotherapy or target specific therapies for cancer patients in clinic are either perfusion or oral administration. At the end, therapeutical molecules reach the tumor through the blood vessels. Although many PDTs-based anti-cancer drug screening models have been established, the PDTs were directly treated in culture in most of these models. In this study, we established a vascularized model to mimic the tumor microenvironment in vivo. Our results revealed that neovascularized vessels could indeed infiltrate into PDTs (Figure 3E, Figure S1), which is in agreement with the previous report [31]. In addition, we have further demonstrated that endothelial cells from the PDTs infiltrated into the surrounding tumor environment and grew rapidly to form a vascular network. It is worth noting that from the same endothelial cells, the neovascularization inside the PDT was not well developed (Figure 4A,B), whereas the surrounding vascular network in the hydrogel had progressed significantly (Figure 4D). This observation could reflect the real situation in vivo where the tumor with an immature vasculature inside was surrounded by the normal microenvironment with well-organized and evenly distributed blood vessels. Finally, we have successfully shown that the vessels neovascularized in the fibrin could indeed be connected with those neovascularized in the PDTs (Figure 5E,F and Supplementary Figure S3). The micro-scale dimension and easy control of fluid make a microfluidic device a perfect partner to 3D cell culture, thus allowing for the creation and mimicking of the dynamic in vivo microenvironment for drug screening [32,33,34]. Since the normalization of the tumor vasculature could be helpful to optimize the current clinical treatment [35], our model would thus be of great interest to be connected with a microfluidic device for drug screening.

## 5. Conclusions

In this study, we have formed patient-derived tumoroids from non-small-cell lung cancer in four days which could be further vascularized and connected to a prevascularized fibrin hydrogel to mimic the real vascular network involved inside the tumor and its microenvironment. This model could be immediately used to test conventional therapies such as radiotherapy, chemotherapy, immunotherapy or virotherapy for personalized medicine, or could be further implemented in a microfluidic device to perform drug screening for personalized medicine.

## Data Availability

Not applicable.

## Supplementary Materials

The following supporting information can be downloaded at: https://www.mdpi.com/article/10.3390/biomedicines10051103/s1, Figure S1: 3D-reconstruction of vascular network connection between vascularized microenvironment and vascularized PDT; Figure S2: Infiltration of endothelial cells from PDT to fibrin gel; Figure S3: 3D-reconstruction of one vessel developed by both GFP and RFP labeled endothelial cells.
