# Supplementary material for: Vascularization of Patient-Derived Tumoroid from Non-Small-Cell Lung Cancer and Its Microenvironment
Source: Biomedicines. 2022 May 10;10(5):1103. doi: 10.3390/biomedicines10051103 (PMC9138465; doi:10.3390/biomedicines10051103)
Supplement: Supplementary file 1 [file biomedicines-10-01103-s001.zip › biomedicines-1684431-supplementary.pdf]

biomedicines

MDPI

Supplementary Data

Vascularization of Patient-derived Tumoroid from non-small-cell lung cancer and its microenvironment

*Author(s), and Corresponding Author(s)**

*Joseph Seitlinger, Anas Nounsi, Ysia Idoux-Gillet, Eloy Santos Pujol, Hélène Lê, Erwan Grandgirard, Anne Olland, Véronique Lindner, Cécile Zaupa, Jean-Marc Balloul, Eric Quemeneur, Gilbert Massard, Pierre-Emmanuel Falcoz, Guoqiang Hua and Nadia Benkirane-Jessel**

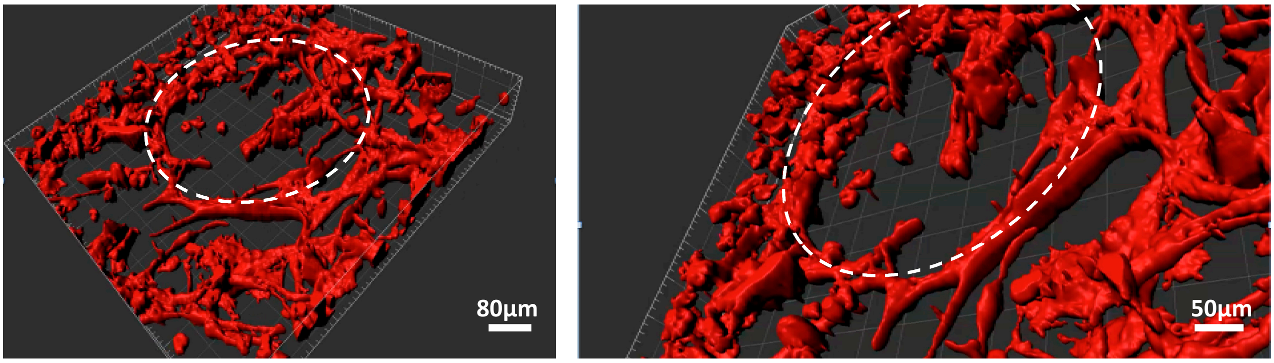

Supplementary Figure S1. 3D-reconstruction of endothliel cell infiltration from vascularized microenvironment to PDT. The dotted lines delimit the PDO, microvessels in red.

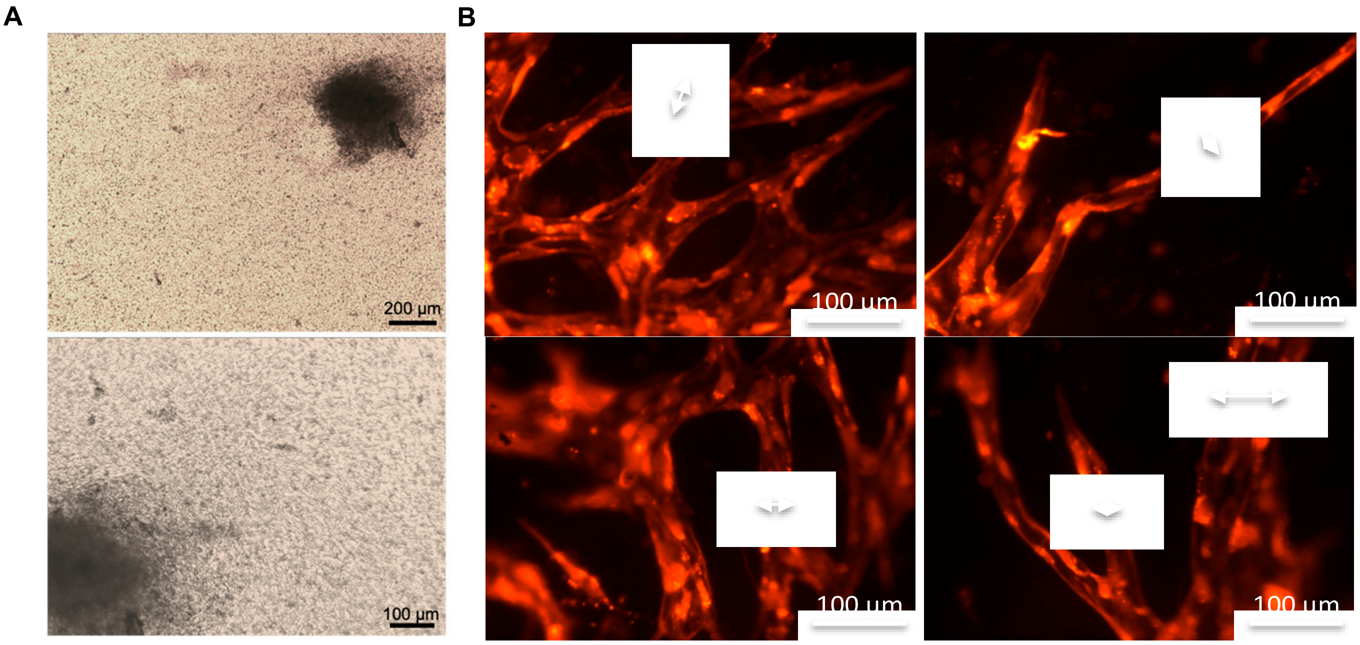

Supplementary Figure S2. Infiltration of endothelial cells from PDT to fibrin gel. (**A**) Bright-field image showing the PDT and the surrounding fibrin gel. (**B**) Measurement of microvessels developped in the fibrin gel.

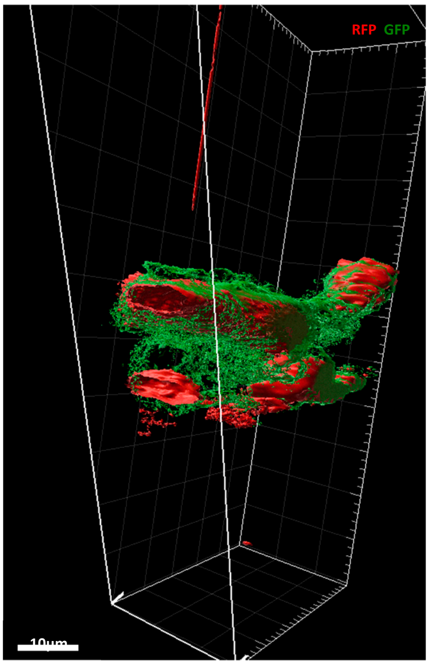

Supplementary Figure S3. 3D-reconstruction of one vessel developed by both GFP and RFP labeled endothelial cells.
